# COVID-19 measures implemented for nursing home staff and their perspectives on the importance of the measures: A qualitative study

**DOI:** 10.1016/j.ijnsa.2023.100163

**Published:** 2023-11-04

**Authors:** Ylse van Dijk, Lisa Sanne van Tol, Willem Pieter (Wilco) Achterberg, Sytse Ulbe Zuidema, Sarah Ingeborg Margit Janus

**Affiliations:** a Department of Primary and Long-term Care, University of Groningen, University Medical Center Groningen, Groningen, The Netherlands; b Department of Public Health and Primary Care, Leiden University Medical Center, Leiden, The Netherlands

**Keywords:** Qualitative research, Staff management, Decision-making, COVID-19, Infection control, Nursing homes, Staff, Long-term care

## Abstract

**Background:**

Measures against COVID-19 in nursing homes affected not only clients but also staff. However, staff perspectives on the importance of these measures remain underexplored.

**Objective:**

To investigate measures related to staff during the COVID-19 pandemic, staff perspectives of important measures and the involvement of staff in deciding on these measures.

**Design:**

A qualitative study.

**Setting(s):**

We analysed minutes of nursing home outbreak teams in the Netherlands and conducted group meetings with Dutch nursing home staff in different positions, prioritizing measures and discussing staff’ involvement in deciding on the measures. Participants were recruited purposefully.

**Participants:**

The minutes of 41 nursing home organizations were collected during March–November, 2020. Four group meetings were organized in the same period, each with 5 to 7 participants, resulting in 23 participants.

**Methods:**

The meeting minutes were analysed using qualitative content analysis, whereas reflexive thematic analysis was used for the group meeting data. The group meetings were conducted online and structured by the Nominal Group Technique to discuss the importance of measures for staff.

**Results:**

Measures implemented for staff focused on prevention of COVID-19 transmission, (suspension of) educational activities, testing, additional tasks and staffing capacity, promoting well-being, and other means of support. The implemented measures overlapped with the measures considered important by staff. In addition, staff considered measures on decision-making support and communication to be important. Staff prioritized the measures in the group meetings because they affected their well-being, workforce scheduling, decision-making, or infection prevention. Furthermore, the group meetings revealed that decision-making shifted from mainly implementing national measures to more context-adjusted decision-making in the staff's or clients’ situations.

**Conclusions:**

We showed that although nursing home staff were not always involved in decision-making during the first COVID-19 wave, there was overlap between the measures implemented by the organizations and measures considered important by staff. We suggest that organizations should be encouraged to explore ways of promoting timely involvement of staff in decision-making; for example, through monitoring staff needs and well-being and giving staff more autonomy to deviate from standard measures during future outbreaks or medical emergencies.

**What is already known about the topic**

• The high risk of COVID-19 in nursing homes entailed drastic measures to prevent infection transmission.
• A few studies have focused on experiences of nursing home staff during the COVID-19 pandemic; however, staff perspectives on measures affecting their work remain underexplored.

Alt-text: Unlabelled box

**What this paper adds**

• We found that although nursing home staff were not always involved in decision-making during the first COVID-19 wave, there was overlap between the measures implemented by the organizations and measures considered important by staff.
• Measures relating to decision-making support and communication were found important by staff but were not listed among the implemented measures, emphasizing the importance of these particular measures in future pandemics.
• Decision-making in the present study shifted from mainly implementing national guidelines to more context-adjusted decision-making in the staffs’ or clients’ situations.

Alt-text: Unlabelled box

## 1 Background

The high risk of SARS-CoV-2 (COVID-19) transmission in nursing homes, attributed to communal living, personal care necessitating close contact, and clients’ frailty, required management to institute drastic measures to prevent infection transmission (Davidson and Szanton, 2020; Dykgraaf et al., 2021; van Tol et al., 2022). This also applied to home care provided by nursing homes, as several clients required daily visits, which increased the risk of transmission (Veldhuizen et al., 2021). These measures impacted not only the clients (nursing home residents and home care clients) but also staff (Gray et al., 2021). For example, the number of practitioners’ physical visits to clients’ homes was restricted (Dykgraaf et al., 2021; Snyder et al., 2021; Verbeek et al., 2020), and nursing staff could work with infected clients in only one location or unit to prevent the virus from spreading (Dykgraaf et al., 2021; Jones et al., 2021; van Tol et al., 2021).

This single-site working policy, along with high absenteeism and pre-existing staffing shortages, compounded workforce management issues (Dykgraaf et al., 2021; van Tol et al., 2021). Therefore, several organizations had to employ temporary staff, non-healthcare nursing home staff, or army medical staff (Fernandes et al., 2021; Gray et al., 2021; Jones et al., 2021). Despite these efforts to boost staff, many studies reported increased workloads and fear of becoming infected or spreading the virus, as well as a higher emotional burden among nursing home staff (Gray et al., 2021; Hendricksen et al., 2022; van Dijk et al., 2022). Although the experiences of nursing home staff during the COVID-19 pandemic have been previously researched (Gray et al., 2021; Sarabia-Cobo et al., 2021; White et al., 2021), specific measures implemented for nursing home staff remain underexplored. Thus, an overview of the implemented measures could advance understanding of the impact of COVID-19 on working procedures for nursing home staff. Specifically, it could provide valuable insights into how staff were supported during these challenging times, as well as the implementation of measures, ultimately informing policymaking for future pandemics.

Decisions on measures and restrictions in nursing homes were influenced by the need for rapid action to prevent the spread of the SARS-CoV-2 virus, which was largely unknown at the time. This situation resulted in top-down decision-making (Behrens and Naylor, 2020; Dykgraaf et al., 2021). The first wave of COVID-19 infections in the Netherlands lasted from February toMay 2020, and the second wave lasted from October 2020 toJanuary 2021 (Rijksoverheid, 2021). During the first wave, Dutch nursing homes instituted COVID-19 outbreak teams to optimize communication, review infection control practices, create a centralized process for monitoring staff and clients, implement protocols for staff, and formulate an action plan for preventing and managing outbreaks (Schols et al., 2020). COVID-19 outbreak teams in Dutch nursing homes consisted mainly of management, medical staff, support services, and communication specialists. Some included nursing staff and resident representatives (van Tol et al., 2022).

However, various studies reported that nursing home staff experienced difficulties applying COVID-19 measures (Hendricksen et al., 2022; Snyder et al., 2021) and wanted to be involved in decision-making (Rutten et al., 2021). Staff involvement in decision-making might improve compliance with introduced measures (Brito Fernandes et al., 2021). To prepare nursing homes for future outbreaks or medical emergencies, it is important that measures fit the needs of staff and their daily working procedures (Hendricksen et al., 2022; Pollock et al., 2020; Snyder et al., 2021). Furthermore, to ensure successful interventions, it is important to know how psychosocial support extended to nursing home staff during the COVID-19 pandemic was actually received (Pollock et al., 2020; Sarabia-Cobo et al., 2021; Soklaridis et al., 2020). However, staff perspectives on measures implemented during an outbreak had not been investigated at the time of this study.

We conducted a qualitative study to acquire insight into the following areas: (1) measures implemented for Dutch nursing home staff during the COVID-19 pandemic, and (2) measures considered important by staff and their involvement in decisions relating to these measures. These perspectives were intended to advance knowledge on how nursing homes can support staff and prepare for future outbreaks or medical emergencies.

## 2 Methods

### 2.1 Research design

As part of a large national multicenter study, minutes were collected from the COVID-19 outbreak teams of 41 nursing homes in the Netherlands from March 2020-November 2021 (van Tol et al., 2021). Within this sub-study, the importance of the implemented measures was discussed with different employees at group meetings held in June, July, September, and November 2020. A positivist Grounded Theory (Merriam and Tisdell, 2015) was adopted to derive meaning from the two data types and consequently build a theory about the specific situation of working in a nursing home during COVID-19 . Hypotheses to be tested were derived from the data and not set out at the beginning of the study. Qualitative content analysis was used for the minutes data (Vaismoradi et al., 2013), whereas reflexive thematic analysis was used for the group meeting data (Willig and Rogers, 2017).

### 2.2 Minutes of COVID-19 outbreak team meetings

Recruitment and procedures applied in the multicenter study are described in more detail elsewhere (van Tol et al., 2021). Briefly, the minutes were compiled weekly and coded under topics by a group of researchers. In the current study, a subset of the data was extracted, with text units coded under the main topic “staff” along with related subtopics. These subtopics were prevention of COVID-19 transmission, (suspension of) educational activities, testing, additional tasks and staffing capacity, promoting staff well-being, and other means of support. Text units between March (week 10) and November (week 47) 2020 were used.

For the analysis, two researchers assigned text units coded under the topic of “staff” to one of the subtopics. A text unit is a piece of text of one to a few sentences from the minutes that relate to one measure (van Tol et al., 2022). Subsequently, all text units coded under the subtopics were checked. Text units that did not match the scope of this study, such as those focusing on hygiene, supply, user instructions, and technical specifics of personal protective equipment; and unclear text units were excluded. Applying qualitative content analysis, we reported both quantitative aspects (the number of text units per topic each month) and qualitative aspects (thematic descriptions) of the data.

### 2.3 Group meetings

For the group meetings, staff from the nursing homes who participated in the multicenter study were recruited. Using a purposive sampling strategy, five to seven employees from different organizations that were involved in providing care, executing administrative tasks, or managing human resources were included in each group meeting. Care positions in Dutch nursing homes include registered nurses, nurse assistants, physicians, and other medical practitioners (e.g., physiotherapists, psychologists, and occupational health physicians) (Hoek et al., 2003; Rutten et al., 2021). Management positions in this sector include the board of directors, members of work councils and team/care/location managers (Zorginstituut Nederland, 2021).

For the group meetings, a nominal group technique (Van de Ven and Delbecq, 1972) was used to discuss measures that staff considered important. The nominal group technique is a structured group process that avoids typical problems encountered by traditional interacting groups, such as dominant speakers and “the focusing effect where groups pursue a single train of thought for a long period” (Gallagher et al., 1993, p. 77). According to this technique, this part of the study consisted of three phases: a preparatory assignment; a group meeting; and the closing assignment, which was not analyzed for this study (see Supplementary Text 1).

The group meetings were conducted using Zoom video conference technology and were led by two moderators with expertise in conducting qualitative research. The moderators were both women and work as fulltime researchers, which positions them as independent interviewers. Each group meeting lasted two hours. The audio recordings were used to transcribe the data. During transcription, identifying information was left out. The transcripts were uploaded into the coding software, Atlas TI.

For the analysis, the transcripts of the first two group meetings were read by two researchers to identify the themes discussed. Using this inductive method, the researchers generated a coding frame and coded the data from these two meetings independently. Subsequently, the coding frame and coded text units were discussed during consensus-building meetings. The remaining two group meetings were coded by one researcher using the generated coding frame, and the codes were discussed with the other researcher. With the addition of new data, new themes and codes were added to the coding frame. Additionally, the coding frame and all coded group meetings were discussed with a third researcher until consensus was reached.

### 2.4 Ethics

Protocols for both the multicenter study (van Tol et al., 2021) and the group meeting sub-study were approved by a local medical ethical committee. Participants at the group meetings provided written consent and approved video recordings of the group meetings.

## 3 Results

### 3.1 Measures affecting nursing home staff derived from the minutes of COVID-19 outbreak team meetings

#### 3.1.1 Quantitative description

**Fig. 1:**
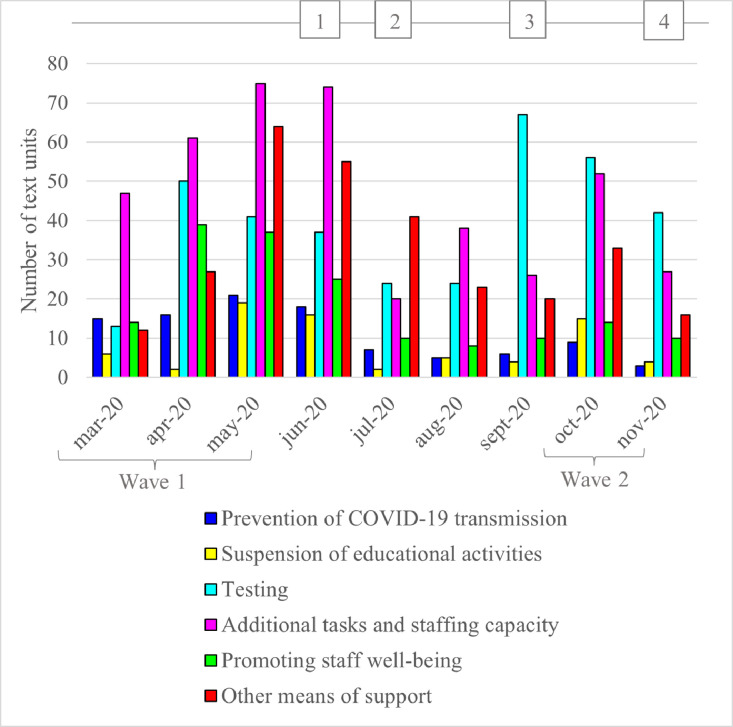
Number of staff-related text units per topic (per month) described in the minutes of COVID-19 outbreak teams meetings at the time of the first and second wave of COVID-19 infections (February–May 2020 and October 2020–January 2021). The numbers in squares represent the timing of the group meetings.

The extracted subset of the multicenter study (van Tol et al., 2021) comprised 1982 text units*.* After excluding and recoding text units (see the Methods section), we obtained 1405 text units, which were coded under: prevention of COVID-19 transmission, suspension of educational activities, testing, additional tasks and staff capacity, promoting staff well-being, and other means of support (Fig. 1). In each topic, the number of text units followed a slightly different pattern over time and in relation to the waves of COVID-19 infections (Fig. 1).

#### 3.1.2 Thematic description

##### 3.1.2.1 Prevention of COVID-19 transmission

Measures were implemented to minimize the risk of COVID-19 transmission in nursing homes (Fig. 1), which limited the mobility of care staff. For example, care staff could work for only one employer or at one location/unit. This stipulation applied to all care staff, apart from practitioners. Practitioners and administrative and facilitating staff were asked to work from home. Measures that prohibited certain employees from being on-site were lifted for groups, such as physiotherapists, hairdressers, and pedicurists, from May 2020 onward, according to the minutes. However, office staff continued to work from home during the study period.

With the decreasing incidence of infection, some organizations implemented less rigorous isolation measures, allowing staff to work at another location as long as they worked at infection-free locations. However, some organizations did allow staff working at an infected location to work at other locations, provided that they wore protective equipment and monitored themselves regularly for symptoms. Another option was to allow staff to work at different locations during the week but at only one location each day. For home care staff, specific routes covering only infected clients were mapped.

*“Home-care clients with symptoms or a confirmed COVID infection are placed on a COVID route to prevent transmission to other clients and outbreaks. . . . A fixed team of staff is working on the routes.” (Organization XO, April 2020)*

##### 3.1.2.2 Suspension of educational activities

This topic covered staff education and the prolongation of certification for specific care or organizational competencies. During the first COVID-19 wave (Fig. 1), almost all educational activities were suspended. Consequently, it became necessary to delay certification, for example, for resuscitation or emergency responses, with or without online training to keep staff qualified during the suspension. After the first wave and during the second wave, organizations decided on necessary training to be provided on- site. Limitations were set on group size, and distancing measures were implemented for on-site educational activities. Also, online possibilities were explored by organizations. These measures also applied to team meetings organized for clinical staff from different locations.

##### 3.1.2.3 Testing

Several changes in testing policies for nursing home staff were made over time, depending on the availability of testing material and according to changes in national policies. When new developments relating to testing emerged, like antibody/antigen tests, they were considered or pilot-tested by the organizations.

During the period March–July 2020, when there was an increased incidence of COVID-19 cases in the Netherlands and testing material supplies were limited, the national public health service set up dedicated telephone lines to make appointments for testing healthcare staff. At the start of each wave, only staff delivering direct care to clients with COVID-19 were allowed to use this line. Because of insufficient testing capacity within the public health service and long processing times for the test results, organizations established their own testing facilities. Additionally, some organizations themselves introduced contact tracing within the work environment.

Organizing available staff to perform these tasks and making arrangements with a lab to process the tests became necessary. However, testing facilities were often regionally organized; some of the nursing homes could use the testing facilities of other organizations like hospitals.

When more tests became available during the first wave, the previously established telephone lines were opened up for other staff, such as cleaners, and later for the families of staff. Changes in the test policy were communicated by the organizations to staff via checklists. In late October 2020, sufficient test material was available, and healthcare staff underwent preventive testing when there was a COVID-19 case at the unit. Once an employee had been tested through the public health service, it was critical that the test result be communicated quickly to the organization to initiate contact tracing immediately. The question of whether the organization or the employee should request the test and receive the test result posed a dilemma, as it was faster for the organization to organize this procedure while also making the organization aware of the medical conditions of their staff. However, this could contravene European privacy laws.

*“It has been asked whether the organization or supervisor could receive a copy of the test results. In this way, contact tracing can be started immediately.” (Organization XB, July 2020)*

##### 3.1.2.4 Extra tasks and staffing capacity

The workload of nursing home staff increased because of extra care or tasks related to COVID-19. Examples included answering questions posed by staff about COVID-19, extra on-call shifts, guiding and informing visitors, and performing contact tracing and testing. Furthermore, extra shifts were required at night because of the extra time taken for staff to change and put on personal protective equipment.

To ensure continuity of care during the pandemic, solutions to staffing shortages were urgently needed. These solutions, covered under this topic, included the deployment of (a) staff with mild symptoms, (b) non-care staff, and (c) external staff.

###### 3.1.2.4.1 Staff with mild symptoms

In urgent staffing shortages, organizations decided to deploy staff with mild COVID-19 symptoms/infection or those awaiting their test results in COVID-19 or normal units.

###### 3.1.2.4.2 Non-care staff

To counter staffing shortages during the pandemic, organizations sometimes required office staff with a care background or facilitating staff to support nursing staff. Exchanges of staff between different locations or even different organizations were also implemented to maintain staffing capacity.

###### 3.1.2.4.3 External staff

In addition to internal solutions, the organizations made efforts to recruit external staff to counter staffing shortages. First, organizations asked retired staff to return to work. Second, family members of clients with care backgrounds were asked to help out. Third, flex workers, freelancers, and temporary agency workers were deployed to fill scheduling gaps and to work in the COVID-19 units. Fourth, organizations offered trainees and medical interns contracts to work on-call or permanently. Fifth, they availed of support provided through national initiatives: volunteers, hospitality industry workers, and army medical staff. Volunteers who had worked at the nursing homes before the pandemic were not asked because of their age and vulnerability to COVID-19. During the recruitment process, because physical interactions were minimized, job applications and introductions occurred online.

##### 3.1.2.5 Promoting staff well-being

This topic included exceptions made for vulnerable employees and the extension of psychosocial support and physiotherapy to promote staff well-being.

###### 3.1.2.5.1 Exceptions for vulnerable employees

Organizations came up with ways to continue deploying vulnerable staff to maintain staffing capacity through consultations with occupational health physicians.

*“In consultation with the occupational health physician, there will be a personal risk inventory with vulnerable staff. Depending on the risk, they can be deployed (with personal protective equipment) in units with no infected clients.” (Organization YD, May 2020)*

###### 3.1.2.5.2 Psychosocial support

The minutes showed that the nursing homes undertook different initiatives to extend psychosocial support for their staff. Such support could be extended by internal or external psychologists, spiritual care workers, and team coaches.

*“Deployment of [our] own psychologists is experienced as [being] very useful and psychologists [can] manage it. All staff (nursing home and homecare) can choose between psychologists [from the organization] or external mental healthcare services.” (Organization XZ, April 2020)*

Additionally, digital interventions and ways for colleagues to share experiences (for example, buddy systems) were promoted. More general initiatives included video messages and banners to encourage and thank staff. Organizations supported their staff via intranet, posters, or newsletters. A more proactive communication strategy entailed requesting psychologists to contact staff and ask them what they needed. Other organizations used questionnaires to identify staff needs and monitor their well-being and workloads.

*“We investigated what staff need while working during COVID-19. The results indicate that we need to provide a varied support program because it is hard to find something that suits everyone's needs.” (Organization YG, November 2020)*

###### 3.1.2.5.3 Physiotherapy

Organizations extended physiotherapy when staff developed physical problems while working from home, as well as rehabilitation treatment for those who suffered from enduring physical problems after a COVID-19 infection.

##### 3.1.2.6 Other means of support

This topic describes support other than mental or physical support. Examples are compensation of testing costs and free childcare for care staff.

*“Children's daycare organization did a really great proposal. [Organization's] staff who struggle to get childcare can bring their children to one of their locations for free.” (Organization XZ, March 2020)*

The organizations also implemented measures for facilitating and administrative staff working from home during the pandemic. They listed and provided the equipment needed to work from home (i.e., laptops, chairs, and desks), depending on their budgets and staff needs. The minutes showed that the organizations were planning to have their administrative staff return to the workplace in June 2020. To ensure safety, the number of people in one room/office was determined according to ventilation capacity and room size, and office staff was asked to be at the workplace on fixed days. However, the intended plan for the return of office staff was postponed to January 2021 by many of the organizations.

COVID-19 constrained opportunities for staff to take breaks and summer vacations. It became necessary for organizations to adjust their procedures for taking breaks, while adhering to isolation and distancing measures. Already planned holidays and extra working hours had to be rearranged, and holiday leave was either withdrawn or encouraged according to staff capacities and care demands.

Caution was advised while planning or going on holidays, and vacation areas were permitted according to risk levels. When employees traveled to a medium-risk region/country, they were responsible for their quarantine time. If the risk level changed from low to medium while the employee was on holiday, the quarantine time became the organization's responsibility.

### 3.2 Measures considered important during group meetings: reasons and decision-making

Nursing home staff with different positions participated in the four group meetings (Table 1). The first two group meetings were held after the first COVID-19 wave in the Netherlands. The last two group meetings were held immediately before and during the second COVID-19 wave. Different employees participated in each meeting, with the exception of one employee who participated in meetings 2 and 3. At the meetings, each participant described one measure; however, during the first meeting, three participants described similar measures, which were therefore summarized, resulting in 21 measures obtained from 23 participants.

**Table 1:** Characteristics of participants’ group meetings.

Group meeting	1	2	3	4
Date	June 9, 2020	July 1, 2020	September 2, 2020	November 20, 2020
Number of experts (*N*)	5	7	5	6
**Sex** Male (*N*,%)	1 (0.2 %)	2 (0.3 %)	1 (0.2 %)	2 (0.3 %)
**Age (years)** Median (range)	31 (31–31)^a^	43 (30–56)^b^	49 (45–57)^c^	55 (48–59)^d^
**Working experience (years)** Median (range)	5 (5–5)^a^	13 (1–28)^b^	14 (10–22)^c^	19 (2–27)^d^
**Position**	Member of the nursing homes’ board of directors, nurse, occupational health physician, psychologist, ‘Sustainable medical care’ project coordinator	Healthcare policy advisor, health and safety advisor (twice), human resources advisor, registered nurse, occupational health physician, ‘Care’ project coordinator	Health and safety advisor, human resources business partner, registered nurse, member of the nursing homes’ board of directors, psychologist	Chair of the nursing homes’ board of directors, healthcare manager, healthcare quality advisor, human resources advisor, personal healthcare assistant, member of the work council

*Note* Missing (*N, %)*:.

a (3, 0.6).

b (2, 0.3).

c (1, 0.2).

d (0, 0) *N* = Number.

As shown in Table 2, measures discussed during the group meetings were similar to the implemented measures recorded in meeting minutes of COVID-19 outbreak teams (preventing the transmission of COVID-19, testing, additional tasks and staffing capacity, and promoting well-being). Three new topics of measures were discussed during the group meetings; (the use of personal protective equipment, decision-making support, and communication). Two topics of implemented measures that were listed among COVID-19 outbreak minutes were not discussed during the group meetings; namely, suspension of educational activities, and other means of support.

**Table 2:** Overview of important measures (*N* = 21) according to the group meeting participants (*N* = 23) in June, July, September and November 2020.

Group meeting (month)	Topic of measure	Reasons
**Preventing the transmission of COVID-19**		
Jun	Staff can have only one employer and can work in only one department or unit.	Staff well-being, Workforce scheduling, Infection prevention
Jul	The organization is centralizing COVID-19 care.	Staff well-being, Workforce scheduling
**Suspension of educational activities (**NA)		
**Testing**		
Jul	Staff with mild symptoms (*are allowed to work with personal protective equipment, and they)*are tested as soon as possible.*	Staff well-being, Infection prevention
Sep	A plan of action is available in case staff or clients have symptoms.	Staff well-being
Sep	A clear testing policy is being implemented (e.g., for staff and their family members with symptoms, staff in close contact with someone who has symptoms, and staff returning from high-risk areas).	Staff well-being
**Additional tasks and staffing capacity**		
Jul	Staff with mild symptoms areallowed to work with personal protective equipment,(and they are tested as soon as possible).*	Staff well-being, Workforce scheduling, Infection prevention
Sep	Non-care staff are being trained in order to deploy them in the care process.	Staff well-being, Workforce scheduling
Nov	Staff who have a COVID-19 infection with mild symptoms can work on a COVID-19 unit. These staff need to distance themselves from other colleagues.	Staff well-being, Workforce scheduling
Nov	Staff are scheduled on a COVID-19 unit as follows: five days at work, five days off.	Staff well-being
**Promoting well-being**		
Jun	The nursing home physician and organization's psychologists are extending support to staff working in a department/unit at two fixed moments per week.	Staff well-being, Workforce scheduling
Jun	Vulnerable healthcare staff are approached by the occupational health physician or organization to discuss their employability.	Staff well-being, Workforce scheduling
Jul	The organization is extending psychosocial support to staff.	Staff well-being
Nov	The organization is extending psychosocial support to staff.	Staff well-being
Nov	The organization is extending psychosocial support to staff.	Staff well-being
**Other means of support** (NA)		
**Topic introduced during group meeting (1): The use of personal protective equipment**		
Jul	Staff are required to use personal protective equipment if a client has symptoms or a confirmed COVID-19 infection.	Staff well-being, Infection prevention
Nov	Staff are receiving training in how to use personal protective equipment properly.	Staff well-being, Infection prevention
Nov	Staff can use personal protective equipment preventively.	Staff well-being, Infection prevention
**Topic introduced during group meeting (2): Decision making support**		
Jul	The organization has appointed an ethical committee.	Staff well-being, Decision-making support
Sep	There are rules in place for staff regarding testing, contact tracing, and psychosocial support, which can be adjusted depending on the specific context.	
Sep	Staff are given more autonomy to deviate from measures.	Staff well-being, Decision-making support
**Topic introduced during group meeting (3): Communication**		
Jul	Daily news updates are communicated to staff by the organization and telephone lines are available for questions or complaints.	Staff well-being
Jul	Updates from the organization (about new protocols assisted by videos) are available for staff via a mobile application.	Staff well-being
Nov	The outbreak team is communicating updates for staff on a daily basis (via a ‘corona dashboard’ or by email).	Staff well-being

⁎ This measure contains two different topics and is therefore represented twice in the table. *N* = Number

#### 3.2.1 Reasons for prioritizing measures

The reasons for prioritizing measures, which concerned staff well-being, workforce scheduling, infection prevention, and decision-making support, as shown in Table 2, are described below per topic of measures.

##### 3.2.1.1 Preventing the transmission of COVID-19

Measures preventing the transmission of COVID-19 transmission (Table 2) were not only considered important for infection prevention during the group meetings but also for staff well-being and workforce scheduling.

A participant in the first group meeting mentioned that staff had to choose for one employer or unit when they worked previously for several organizations or units. This had a negative effect on staff well-being, especially when the decision was made by the organization.

*“It really affected staff when a team was redistributed among different units by the organization.” (Group meeting June 2020)*

According to a participant in the second group meeting, working in an infection-free unit could be experienced as more stressful than working in a COVID-19 unit because of the risk of infecting the unit.

With regard to workforce scheduling, staff working in one department/unit resulted in clients seeing familiar faces. Moreover, the centralization of COVID care was considered important to reduce the workload.

*“One of the reasons why we centralized COVID care was to save time. At a COVID unit, staff did not have to change their protective clothing all the time.” (Group meeting July 2020)*

##### 3.2.1.2 Testing

Measures about testing were not only considered important for infection prevention but also for staff well-being and workforce scheduling. That is, measures about testing were expected to relieve uncertainty among staff in the group meetings. The implementation of a quick testing process was foregrounded for ensuring the continuity of essential care tasks.

##### 3.2.1.3 Additional tasks and staffing capacity

Measures about additional tasks and staffing capacity were considered important for staff well-being and workforce scheduling. Training non-care staff to work in a specific unit was related in a group meeting with increased staff well-being since this measure enhanced understanding between non-care and care staff. Other measures were considered important for staff well-being, since they prevented staffing shortages or reduced the workload. Most of the measures concerning additional tasks and staffing capacity were considered important for workforce scheduling as well to ensure the continuity of essential care tasks.

*“There were so many COVID infections in the nursing home where I worked. We had to deploy staff with COVID symptoms because otherwise we did not have enough capacity to provide care.” (Group meeting July 2020)*

##### 3.2.1.4 Promoting well-being

Measures about promoting well-being were considered not only important for staff well-being but also for workforce scheduling. Group meeting participants expected most of the measures to relieve fear or stress. Participants stated psychosocial support was needed because staff not only feared becoming infected themselves but also that clients or their family members would become infected. Moreover, participants mentioned that consultations with medical staff were experienced as important to share experiences and discuss questions

*“It was nice that staff could share their experiences with us [psychologists] and we could answer their questions, as staff got confused by daily changing measures.” (Group meeting June 2020)*

Participants in the second and last group meeting mentioned that staff needed to be invited to register for the support programs rather than asking for help themselves and were positive about using the organization's own psychologists to provide support during the pandemic. They felt it was important that the support matched staff needs and therefore suggested that organizations monitor these.

Regarding workforce scheduling, measures were considered important to prevent staffing shortages and reduce the workload.

##### 3.2.1.5 The use of personal protective equipment

Measures about the use of personal protective equipment were considered important for staff well-being and infection prevention in the group meetings. Although the use of personal protective equipment was mainly considered supportive for staff, negative consequences of wearing personal protective equipment in summer when temperatures were high were also reported.

##### 3.2.1.6 Decision-making support

Measures about decision-making support were considered important to lighten ethical dilemmas about clients’ well-being during COVID-19. The ethical dilemmas discussed at the group meetings were about family visit restrictions and how to handle clients’ wishes (e.g., when staying inside was mandatory). Participants reported that some organizations appointed ethical committees to provide staff with decision-making support because of the life and death dilemmas that they faced daily. In other organizations, professional responsibility was deemed important because deviation from the measures implemented was sometimes warranted. Staff was given more autonomy to deviate from measures and make decisions on the basis of their own knowledge and in discussion with their supervisors and other professionals.

*“Some rules were very clear, but it was difficult to decide whether you could make an exception for a client or not. The possibility of discussing these dilemmas helped staff during these stressful situations.” (Group meeting 2, July 2020)*

##### 3.2.1.7 Communication

Measures about communication were considered important for staff well-being. Clear communication with staff, for example, through (daily) updates via a mobile application, dashboard posts, or email and answering their questions led to a feeling of being supported and prevented panic in the department.

#### 3.2.2 Decision-making

At the last group meeting, participants mentioned that during the first COVID-19 wave, the COVID-19 outbreak team mainly implemented national measures in their organizations. During the second wave, there was more room to customize decisions for both clients and staff according to the participants. National measures were later on changed to policies for care organizations, allowing more customized decisions to be made at nursing home level by local crisis outbreak teams. By then, crisis outbreak teams focused more on receiving input from staff, for example, via managers or supervisors, intranet pages, work councils, e-mail, telephone lines, and questionnaires.

## 4 Discussion

We investigated measures implemented for nursing home staff during the COVID-19 pandemic and identified those that the staff considered important. The implemented measures recorded in the minutes overlapped with the measures considered important by staff during the group meetings. During the group meetings staff described a shift from implementing national measures in the organizations without reference to the local context to more context-adjusted decision-making during the second wave.

During the group meetings, increased workloads were identified as a challenge, which supports the findings of previous studies (Gray et al., 2021; Mojtahedzadeh et al., 2021; White et al., 2021). In the present study, workloads increased because of additional COVID-19-related tasks recorded in the minutes of the COVID-19 outbreak team meetings, such as contact tracing, testing, and guiding visitors. Other measures that we identified were aimed at addressing staffing shortages and psychosocial support for staff. The psychosocial support extended included personal and group approaches (Pollock et al., 2020). The finding that support should also be available for staff who were not working with clients infected with COVID-19 was foregrounded in our earlier study. In this study, we found minor differences in mental health outcomes between those who worked with clients infected with COVID-19 and those who did not (van Dijk et al., 2022).

During the group meetings, participants stressed the importance of actively approaching staff to register in support programs. A previous study also reported the reluctance of care staff to seek help for themselves (Chen et al., 2020). Specifically, this study found that during the COVID-19 outbreak in April 2020, Chinese hospital nurses ignored signs of psychological distress because they worried more about their family than about themselves (Chen et al., 2020). These nurses suggested modes of support that differed from what was extended (Chen et al., 2020). The focus on staff needs is important while preparing for outbreaks, as healthcare organizations tend to focus mainly on preparations relating to medical, hygienic, and organizational aspects of care (Pollock et al., 2020).

Although previous researchers have reported a lack of awareness of staff needs and resources (Hendricksen et al., 2022; Pollock et al., 2020; Rutten et al., 2021; Snyder et al., 2021; White et al., 2021), we showed that organizations monitored staff needs and well-being during the pandemic. Decision-making in the present study shifted from primarily top-down to more context specific in the staffs’ or clients’ situations.

To the best of our knowledge, this is the first study focusing on COVID-19 measures considered important by staff in a healthcare setting. Given our intention to avoid adding to the workloads of participating organizations, we used their existing documents to develop an overview of the implemented measures. The group meetings provided useful context. Moreover, online group meetings enabled us to include staff who held various positions in nursing homes throughout the Netherlands.

## 5 Limitations

This study also had some limitations. First, because of the types of documents used, measures were not described in detail, and the overview of measures was limited to the topics discussed in the meetings. Second, the recounting of earlier experiences is prone to recall bias (Snyder et al., 2021). Third*,* a relatively small number of staff working with infected clients participated in the group meetings. The increased work strain during the COVID-19 pandemic might have impeded care staff or hindered managers from inviting care staff to participate in the group meetings. This may have led to biased findings.

## 6 Conclusions

In this study, we showed that, although nursing home staff were not always involved in decision-making during the first COVID-19 wave, there was an overlap between the measures implemented by the organizations and measures considered important by staff. However, measures relating to decision-making support and communication were an exception, as staff found these measures important, but they were not listed among the implemented measures. We found that organizations were already monitoring the needs and well-being of staff. Organizations should be encouraged to think of ways to involve staff in timely decision-making and to give them more autonomy in certain cases to deviate from standard measures during future outbreaks or medical emergencies.

## Funding

This work was supported by the Dutch Ministry of Health Welfare and Sport (grant number 330526). The sponsor had no further involvement in the study.

## CRediT authorship contribution statement

**Ylse van Dijk:** Formal analysis, Visualization, Writing – original draft, Writing – review & editing. **Lisa Sanne van Tol:** Conceptualization, Formal analysis, Investigation, Writing – review & editing. **Willem Pieter (Wilco) Achterberg:** Conceptualization, Funding acquisition, Writing – review & editing. **Sytse Ulbe Zuidema:** Conceptualization, Funding acquisition, Supervision, Writing – review & editing. **Sarah Ingeborg Margit Janus:** Conceptualization, Formal analysis, Funding acquisition, Investigation, Supervision, Writing – review & editing.

## Declaration of competing interest

The authors declare that they have no known competing financial interests or personal relationships that could have appeared to influence the work reported in this paper.

## Appendix Supplementary materials

Image, application 1

## Footnotes

Supplementary material associated with this article can be found in the online version at doi:10.1016/j.ijnsa.2023.100163.
