# Supplementary material for: COVID-19 measures implemented for nursing home staff and their perspectives on the importance of the measures: A qualitative study
Source: Int J Nurs Stud Adv. 2023 Nov 4;6:100163. doi: 10.1016/j.ijnsa.2023.100163 (PMC11080563; doi:10.1016/j.ijnsa.2023.100163)
Supplement: Supplementary file 1 [file mmc1.docx]

Supplementary Text 1: *Detailed method of group meetings*

| - For the *preparatory assignment*, a list of measures derived from the minutes data was shared with the participants, who were asked to choose the measure they considered most important for staff during the COVID-19 pandemic. This procedure facilitated the incorporation of new measures that were not listed. The participants were also asked which perspective was applied when their chosen measure was decided. - The participants had to send the preparatory assignment to the researchers before commencing the group meeting. - During the *first part of the group meeting*, participants were individually asked to describe the measure that they deemed most important and the reasons for their choice. - During the *second part of the group meeting*, they were asked who was involved in deciding the measure. - Before each part of the group meeting ended, there was time for questions or discussion facilitated by the moderated to elaborate on the measures chosen. - After the group meeting, a *closing assignment* was sent to the participants by e-mail. In this assignment the participants were asked to rate the importance and urgency of the discussed measures on a 5-point Likert scale. |
| --- |
